# Research based on the core pathogenesis in the treatment according to traditional Chinese medicine syndrome differentiation for heart failure with normal ejection fraction

**DOI:** 10.1097/MD.0000000000021663

**Published:** 2020-09-11

**Authors:** Zhiqiang Zhao, Xianliang Wang, Shuai Wang, Ruijuan Zhou, Quan Su, Yu Liu, Tao Cheng, Qing Li, Shanshan Lin, Hua Liu, Jingyuan Mao

**Affiliations:** Cardiovascular Department, First Teaching Hospital of Tianjin University of Traditional Chinese Medicine, Tianjin, China.

**Keywords:** heart failure, randomized controlled trial, traditional Chinese medicine, Yangyin Shuxin Decoction

## Abstract

**Background:**

The incidence of heart failure with normal ejection fraction (HFNEF) is increasing yearly, accounting for approximately half of all heart failure cases. Even after standardized treatment, the patient's prognosis is not good. Therefore, it is necessary to explore new treatment methods for HFNEF. Yangyin Shuxin Decoction, a traditional Chinese medicine prescription from our clinical experience in the treatment of HFNEF, has a potential cardioprotective effect. Preliminary clinical trials have shown that this prescription can improve the quality of life of HFNEF. This prompted us to use more objective indicators to further evaluate whether Yangyin Shuxin Decoction can improve the exercise capacity in HENEF patients.

**Methods:**

This is a single-center parallel randomized controlled trial. The 64 patients who met the inclusion criteria were from the Cardiovascular Clinic. They will be randomly assigned to the treatment group (Yangying Shuxin Decoction combined with standard treatment) or the control group (standard treatment) according to the ratio of 1:1. The course of treatment will be 2 weeks. Both groups were interviewed at the following time points: of at enrollment (V1), and week 2 (V2), week 4 (V3), week 8 (V4), and week 12 (V5) after enrollment. The primary indicator is the peak oxygen consumption (Peak VO2) of the cardiopulmonary exercise test (CPET). Secondary indicators include CPET indicators such as anaerobic threshold oxygen consumption, carbon dioxide ventilation equivalent slope, echocardiographic indicators such as the ratio of mitral peak velocity of early filling to early diastolic mitral annular velocity(E/e′), left atrial volume index (LAVI), left ventricular mass index (LVMI), the peak velocity of tricuspid regurgitation (TR), B-type natriuretic peptide (BNP), New York Heart Association (NYHA) cardiac function grading, and so on. These indicators will be used to evaluate the effect of Yangyin Shuxin Decoction on exercise capacity in patients with HFNEF.

**Discussion:**

At present, it is unclear whether the exercise capacity can be maintained after long-term use of Yangyin Shuxin Decoction. In this study, we will evaluate whether Yangyin Shuxin Decoction can improve the exercise capacity and quality of life of patients with HFNEF. This will provide an objective basis for the therapeutic effect of traditional Chinese medicine on HFNEF.

**Trial Registration:**

This study protocol has been listed in the Chinese Clinical Trial Registry (registration number: ChiCTR-IOR-17014206, http://www.chictr.org.cn/showproj.aspx?proj=24304) on December 28, 2017.

## 1 Introduction

China gradually enters an aging society and the incidence of heart failure with normal ejection fraction (HFNEF) is increasing yearly.^[1]^ These patients account for approximately half of all patients with heart failure (HF).^[2–4]^ Beta-adrenergic receptor blockers, angiotensin-converting enzyme inhibitors (ACEI), angiotensin receptor blockers (ARB), and so on are routinely used to treat HFNEF. However, they have not improved the prognosis and reduced the mortality of patients with HFNEF.^[5–7]^ Patients with HFNEF who received conventional treatment still have problems, such as low exercise capacity and low quality of life.^[8]^ Previous clinical studies^[9–12]^ suggested that traditional Chinese medicine (TCM) had certain efficacy in relieving symptoms, increasing activity capacity, improving quality of life, and other aspects in patients with HFNEF. However, the quality of these studies is poor. Some deficiencies reduce the level of evidence such as large differences in the study population, unclear inclusion/exclusion criteria, non-uniform TCM syndromes, relatively single clinical evaluation indicators, and other problems.^[13]^

Yangyin Shuxin Decoction is a TCM prescription for treating HFNEF. It has the effects of nourishing Yin (Yangyin), promoting blood circulation (Huoxue), and clearing away heat (Qingre). Preliminary clinical trials have proved that it can improve the quality of life of patients with HFNEF.^[14]^ The single drug component of each drug in the prescription has multiple targets to improve the heart and lung functions and increase the exercise capacity. Hence, we planned to use more objective indicators to further evaluate whether Yinyin Shuxin can improve the exercise capacity of patients with HFNEF.

**Figure 1:**
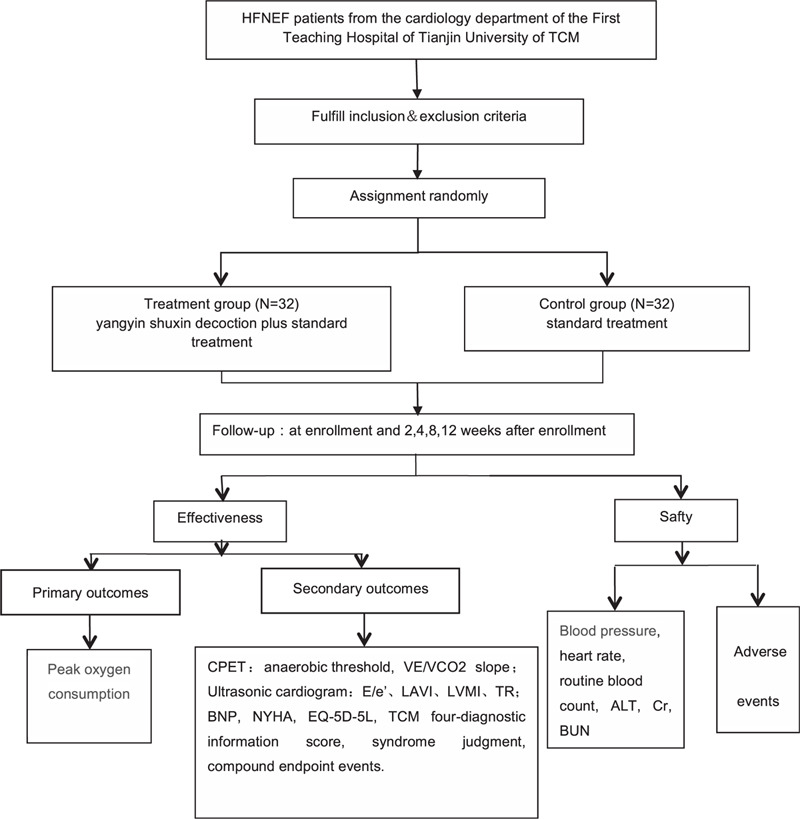
Study flow chart.

We hypothesized that Yangyin Shuxin Decoction could improve the exercise capacity of patients with HFNEF. Moreover, we designed this randomized controlled trial to compare the efficacy of conventional western medicine and Yangyin Shuxin Decoction combined with conventional western medicine on exercise capacity in patients with HFNEF. The relevant design principles and implementation schemes of the randomized controlled trial protocol are as follows (Fig. 1).

## 2 Methods

### 2.1 Study design

This is a single-center, prospective, parallel, and randomized controlled trial. Based on a computer-generated randomized number, 64 patients with HFNEF will be randomly assigned to either the treatment or control group. Patients in the treatment and control groups will be treated with a drug intervention for 2 weeks and followed up for 12 weeks. We will terminate this test when the following situations occur:

1. a serious safety incident occurred during the test;
2. there are major errors in the clinical trial protocol;
3. serious deviations occur so that it is difficult to evaluate the efficacy of the drug; and
4. the project management department cancels the test.

A data monitoring committee will be established, mainly to make interim analysis and assess adverse events. The committee will review the core trial processes and documents, and discuss any amendments to the main study protocol. Any adverse event will be recorded in the CRF and reported to the data monitoring committee in a timely manner. We will provide appropriate compensation for any injured subjects. The Standard Protocol Items: Recommendations for Interventional Trials (SPIRIT) checklist is provided as Additional file 1.

### 2.2 Participants

All of the 64 patients with HFNEF will be enrolled in the Department of Cardiology, the First Teaching Hospital of Tianjin University of TCM. First, the patients will be fully introduced to the benefits and risks of the treatment. Second, the patients need to sign the informed consent on a voluntary basis. Then, the patients will be divided into the treatment (Yangying Shuxin Decoction combined with the standard treatment) or control (standard treatment) groups according to the coding sequence from the pre-set random number table. The inclusion and exclusion criteria are shown in Table 1.

**Table 1:**
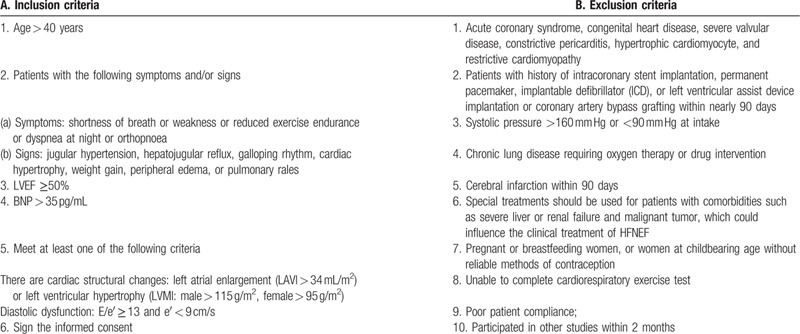
Eligibility criteria.

The follow-up period was 12 weeks. A full physical examination and assessment of adverse events will be performed for all participants. After the patient signs the informed consent form, we will collect general information including demographics, medical history, and concomitant medications. Two clinical controls were performed at the time of enrollment (V1) and week 2 (V2). Indicators include cardiac ultrasound, CPET, BNP, NYHA cardiac function grading, EQ-5D-5L, TCM four-diagnostic information score, syndrome judgment, blood pressure, heart rate, weight, and so on. Blood and urine samples will also be taken. During the follow-up period, the investigator will contact the participants via telephone at weeks 4 (V3), 8 (V4), and 12 (V5) to monitor the patient's blood pressure, weight, NYHA cardiac function, hospitalization/outpatient costs, and compound endpoint events to assess the patients’ quality of life. This program was prepared in accordance with the standard protocol project SPIRIT 13. The specific process of the study is shown in Table 2.

**Table 2:**
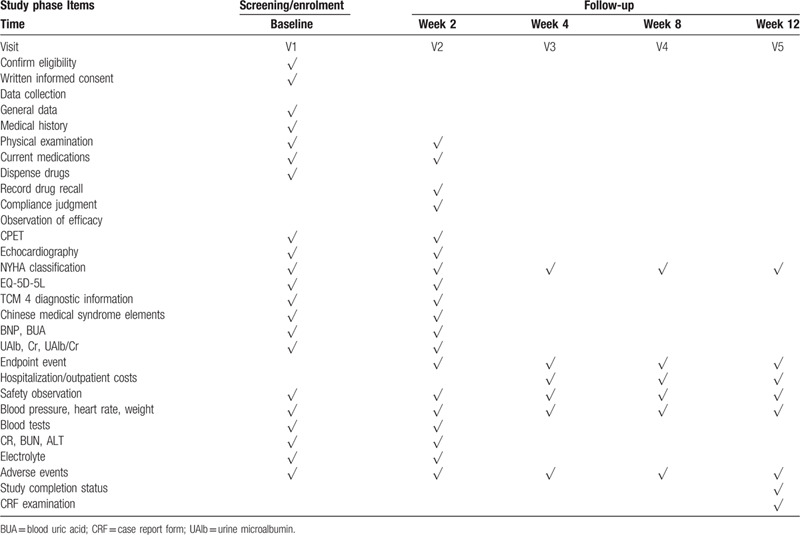
Research access flow chart.

### 2.3 Interventions

Other TCM preparations are not allowed during the washout period for 2 weeks. The patients in the treatment group will be treated with conventional western medicine combined with Yangyin Shuxin Decoction 150 mL, twice a day. All Chinese herbal medicines in Yangyin Shuxin Decoction are from the Department of Pharmacy, the First Teaching Hospital of Tianjin University of TCM. The decocting room is uniformly fried and made into a vacuum package of 150 mL. The remaining decoction will be recycled if the participants withdraw from the test midway. The patients in the control group will receive conventional western medicine treatment. The duration will be 2 weeks. Both groups will be interviewed at the following time points: enrollment (V1), week 2 (V2), week 4 (V3), week 8 (V4), and week 12 (V5) after enrollment.

The TCM theory believes that patients with HFNEF have clinical syndrome characteristics of yin deficiency, blood stasis, and internal heat.^[15]^ Each of the single-drug ingredients in Yangyin Shuxin Decoction includes a variety of compounds such as polyphenols, terpenoids, saponins, and alkaloids that are beneficial to the cardiovascular system. The relevant bioactive ingredients and potential mechanisms are shown in Table 3. These ingredients have the combined effects of improving heart and lung functions, increasing activity tolerance, improving microcirculation, and improving immunity and body antioxidants in patients with HFNEF.

**Table 3:**
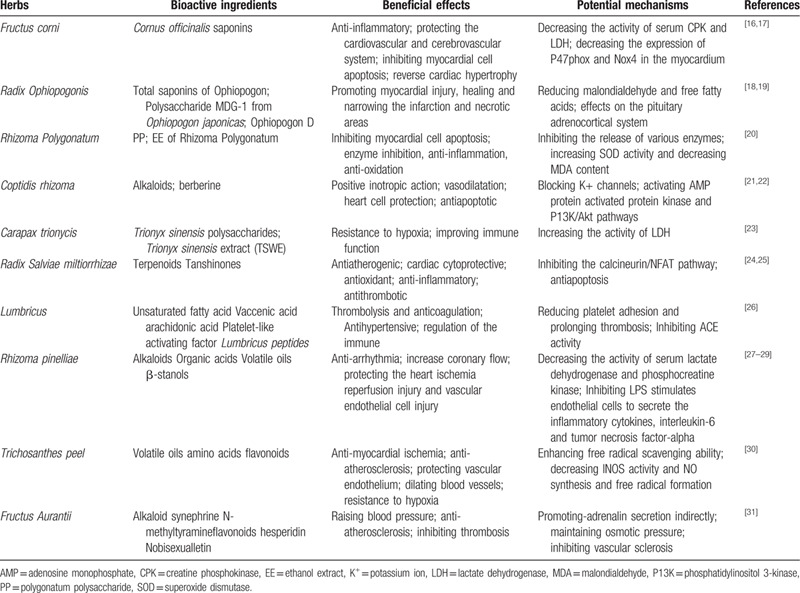
Cardiovascular effects and potential mechanisms of active ingredients.

Western medicine standard treatment plan is implemented according to the “China Heart Failure Diagnosis and Treatment Guide 2014”^[32]^ and “2016 ESC Guidelines for the diagnosis and treatment of acute and chronic heart failure.”^[33]^ The Western medicine standard treatment includes health education for patients to help them establish a heart-healthy lifestyle (such as salt restriction, water restriction, weight monitoring, physical exercise, smoking cessation, alcohol withdrawal, and so on) and medication guidance and emergency management instructions. In addition, other TCMs for the treatment of cardiovascular diseases should be avoided.

### 2.4 Outcomes

#### 2.4.1 Primary outcome

The change of Peak VO2 in cardiopulmonary exercise test.

Secondary outcomes:

- anaerobic threshold and ventilatory equivalent for carbon dioxide (VE/VCO2) slope detected in the cardiopulmonary exercise test.
- E/e′, LAVI, LVMI, and TR detected with ultrasonic cardiogram.
- BNP, NYHA cardiac function grading, EQ-5D-5L, TCM four-diagnostic information score, syndrome judgment, compound endpoint events, and so on.

#### 2.4.2 Security outcomes

Vital signs, some laboratory tests, and adverse events are considered as safety outcomes. Vital signs, including blood pressure and heart rate, routine laboratory tests (routine urinalysis, routine blood test, and hepatic and renal functions), and electrocardiograms, and the adverse events will be documented at each visit.

### 2.5 Patient and public involvement

The patients or the public were not involved in the design or in conducting, reporting, or disseminating our research.

### 2.6 Sample size

The sample size is computed based on the literature “Effect of If-channel inhibition on hemodynamic status and exercise capacity in HF with preserved ejection fraction: a randomized trial,”^[34]^ peak VO2 on day 7 is 3.0 ± 3.6 mL/kg/min in the treatment group and 0.4 ± 2.7 mL/kg/min in the control group. Sixty-four patients will be recruited with a single-sided alpha of 0.05, a power (1 − *β*) of 0.90, and a dropout rate of 20%. The calculation formula is as follows^[35]^: 
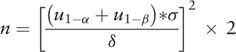


### 2.7 Blinding

Blinding the researchers responsible for the implementation and patients included is not possible due to the particularity of dosage forms. The investigators are responsible for distributing the drugs. To ensure the reliability of the test, the personnel and statistical experts performing the outcome index evaluation will be blinded. All research team members were instructed not to communicate with the participants regarding their allocation.

### 2.8 Data collection and management

To assess the patients’ health status since the last visit and HFNEF-related re-hospitalization or unplanned medical conditions throughout the study period, the two groups will be contacted every 2 weeks or a month. All original data will be observed directly by clinical researchers and documented completely and timely in the case report form (CRF), including the reasons why patients cannot participate. To ensure the reliability of data, the CRF will be entered by the double-entry method. All errors will be crossed out and corrected and signed by the corresponding investigator. All these data will be locked in a separate cabinet. Only authorized investigators are permitted to access this information.

### 2.9 Statistical analysis

SPSS23.0 statistical analysis software will be used to calculate the test data, and descriptive statistics will be conducted for all the data. For the differences between the test groups, chi-square test will be used for the counting data. The *t* test will be applied when normality (and homogeneity of variance assumptions) is satisfied, otherwise the rank sum test will be used.

The number of screened patients and reasons for exclusion will be reported, as well as protocol violations and reasons. An effectiveness analysis will be conducted using the randomized populations. Participants who have received treatment but there is no valid evaluation data will be considered as missing and will be included in the effectiveness analysis.

## 3 Discussion

To the best of our knowledge, this is the first randomized controlled trial using cardiopulmonary exercise test indexes to evaluate the impact of TCM on the exercise capacity of patients with HFNEF. Cardiopulmonary exercise test indicators (including peak VO2, anaerobic threshold, VE/VCO2 slope, and so on) are taken as the main indicators, and combined with echocardiography, BNP, NYHA cardiac function grading, generic EQ-5D-5L, and other indicators normally used to evaluate the prognosis of patients with HFNEF, to comprehensively evaluate the prognosis of HFNEF patients. Peak VO2, the main efficacy indicator, is an important indicator to evaluate the aerobic work ability of the human body. Not only can it be used to evaluate the severity of HFNEF and the effect of cardiac rehabilitation training, but also closely related to the long-term prognosis of patients.^[36–38]^ Peak oxygen consumption is also an effective and practical evaluation index of aerobic capacity and has good retest reliability.^[39]^ The anoxia threshold, a secondary therapeutic index, can reflect the body's potential to tolerate load. It is usually measured by sub-maximum exercise and is rarely affected by subjective and objective factors. It can sensitively show the balance of tissue oxygen supply and demand and accurately evaluate the anoxia metabolic capacity and cardiopulmonary function during exercise.^[40]^ In addition, Nedeljkovic^[41]^ observed that, in HFNEF patients undergoing cardiopulmonary exercise test combined with exercise load ultrasound, the equivalent slope of carbon dioxide ventilation (VE/VCO2 slope) was an independent predictor of HFNEF prognosis. At the same time, the echocardiographic index E/e′ was used to evaluate cardiac diastolic function, left atrial pressure, and left ventricular filling pressure volume curves, which were estimated by this method to be very close to those of the invasive catheterization method; and it is not affected by ejection fraction and cardiac arrhythmias and has now been designated as one of the main ultrasonic screening parameters of HFNEF by the guidelines.^[42]^

In addition, during the follow-up period of 3 months, patients’ clinical conditions, especially regarding the re-hospitalization rate and the outpatient and inpatient costs will be tracked to comprehensively evaluate the patients’ exercise capacity and quality of life. At present, it is unclear whether the exercise capacity can be maintained after the long-term use of Yangyin Shuxin Decoction. Even so, this study will provide valid data for TCM to improve the exercise capacity of patients with HFNEF. Although it cannot fully explain the multi-linked interventional and pharmacological mechanism of TCM decoction on HFNEF, we can still use it as an intervention method and explore the mechanism from the perspective of pathophysiology in the future, because the fact that it can improve exercise tolerance in patients with HFNEF has been proven.

In conclusion, this is a single-center parallel randomized controlled trial. We will evaluate whether Yangyin Shuxin Decoction can improve the exercise capacity and quality of life of patients with HFNEF. This will provide an objective evidence for the therapeutic effect of TCM on HFNEF.

## Acknowledgments

We thank all the participants in our study. We would like to thank Editage (www.editage.cn) for English language editing.

## Author contributions

**Data collection:** Shuai Wang, Ruijuan Zhou, Yu Liu.

**Project administration:** Zhiqiang Zhao.

**Recruitment patients:** Zhiqiang Zhao, Quan Su, Tao Cheng, Qing Li, Hua Liu.

**Treatment:** Zhiqiang Zhao.

**Trial design:** Jingyuan Mao, Zhiqiang Zhao, Xianliang Wang.

**Writing – original:** Zhiqiang Zhao, Qing Li, Shanshan Lin.

**Writing – review & editing:** Zhiqiang Zhao, Xianliang Wang, Jingyuan Mao, Zhiqiang Zhao.
